# Self-sensing of temperature rises on light emitting diode based optrodes

**DOI:** 10.1088/1741-2552/aaa56d

**Published:** 2018-01-25

**Authors:** Fahimeh Dehkhoda, Ahmed Soltan, Nikhil Ponon, Andrew Jackson, Anthony O’Neill, Patrick Degenaar

**Affiliations:** 1 School of Engineering, The University of Edinburgh, Edinburgh EH9 3JL, United Kingdom; 2 School of Engineering, Newcastle University, Newcastle upon Tyne NE1 7RU, United Kingdom; 3 Institute of Neuroscience, Faculty of Medical Sciences, Newcastle University, Newcastle upon Tyne NE2 4HH, United Kingdom; f.dehkhoda@ed.ac.uk; ahmed.abd-el-aal@newcastle.ac.uk; nikhil.ponon@newcastle.ac.uk; andrew.jackson@newcastle.ac.uk; anthony.oneill@newcastle.ac.uk; patrick.degenaar@newcastle.ac.uk

**Keywords:** temperature sensor, LED, optical stimulation, optogenetics

## Abstract

*Objective*. This work presents a method to determine the surface temperature of microphotonic medical implants like LEDs. Our inventive step is to use the photonic emitter (LED) employed in an implantable device as its own sensor and develop readout circuitry to accurately determine the surface temperature of the device. *Approach*. There are two primary classes of applications where microphotonics could be used in implantable devices; opto-electrophysiology and fluorescence sensing. In such scenarios, intense light needs to be delivered to the target. As blue wavelengths are scattered strongly in tissue, such delivery needs to be either via optic fibres, two-photon approaches or through local emitters. In the latter case, as light emitters generate heat, there is a potential for probe surfaces to exceed the 2 °C regulatory. However, currently, there are no convenient mechanisms to monitor this *in situ*. *Main results*. We present the electronic control circuit and calibration method to monitor the surface temperature change of implantable optrode. The efficacy is demonstrated in air, saline, and brain. *Significance*. This paper, therefore, presents a method to utilize the light emitting diode as its own temperature sensor.

## 1. Introduction

Implantable devices are becoming increasingly important in clinical practice. Biosensors, pacemakers and prosthetics such as visual prosthesis [1] may all utilise optoelectronics. These can be classified into two primary applications: opto-electrophysiology [2] and fluorescence sensing [3]. The former allows control and recording of electrical activity in tissue. The latter can be used to explore chemical changes in cells or their environment. The advent of optogenetic reporters, such as green fluorescent protein in the 1990s [4], has allowed imaging of the presence (or lack) of specific cell types. More recently functional derivatives (e.g. GCaMP-6) [5] allow for the optogenetic imaging of calcium flows and electrical function. Similarly, the advent of optogenetic membrane channels has allowed for *in situ* genetic modification of light sensitivity in cells. Such optogenetic stimulation can use either channelrhodopsins—light-sensitive cation channels [6], halorhodopsins—light-sensitive ion pumps [7], or melanopsins—light sensitive G-protein coupled receptor systems [8]. Each has their own relative advantages. A review of the field has been written by Barrett *et al* [9].

What is common to all these approaches is that they require moderate to high-intensity irradiation [10]. Furthermore, the optically active cores in all of these proteins are typically sensitive to UV to green wavelengths. UV is undesirable *in vivo*, but even blue and green wavelengths are strongly scattered by neural tissue [11]. This means there is a requirement for light delivery in close proximity to the stimulus target. This can be achieved by one of two methods: Light can be generated at some distance and then guided to the local position using optical confinement (e.g. optic fibre or waveguide) until the point of delivery [12–14]. Alternatively, light can be produced locally on a penetrating or otherwise implantable device incorporating a microphotonic element [15–17]. The latter method’s advantage is being able to electronically multiplex multiple outputs leading to a small cable. Both methods are being explored, and there are pros and cons for each. In the case of optical delivery to the target, it can be challenging to multiplex large numbers of independent signals in a single system. Furthermore, the light generator will still be implantable and will still generate heat, albeit perhaps from a domain that can better tolerate thermal load. In the case of microphotonic generation close to the target, the key issue is the formation of localized hot spots close to the biomedical device surface.

It was shown by Stujenske [18] in 2015 that high radiance light absorption from optic fibre emission can cause localised tissue heating. If the emission source is microphotonic element, then there is additional potential for heating. McAlinden *et al* in 2013 proposed from modelling that there could additionally be a few degrees of temperature rise depending on how the microphotonic element is driven [17]. The current literature is not entirely clear on what long-term effects could result from such temperature increase. In 1989, Lamanna *et al* [19] performed acute experiments on anaesthetized rats and suggested that temperature should not increase more than 1 °C. This threshold has since been used by over 60 other studies [17, 20]. However, the 1 °C limit is actually a suggestion rather than a rule as they did not see a negative effect on the transient increase below 5 °C. They also performed their experiments in tissue which was unnaturally cold (2 °C lower than the body) due to the anaesthetic. More recently in 2012, Opie *et al* [21] looked at *ex vivo* tissue and determined 38.7 °C as the threshold at which damage would begin to occur in the retina. But, perhaps the retina is less prone to thermal damage than brain tissue due to its various mechanisms to protect itself from phototoxicity. Perhaps the most conclusive work was by Matsumi *et al* [22], who studied the effect of insertion of heated probes into live non-human-primate brain. They found that thermal damage occurred after 44 °C, and suggested a thermal limit of 43 °C—i.e. dT  =  6 °C. These results match the earlier results of Lamanna *et al* [19]. Fujii *et al* [23] come to the same 43 °C conclusion with guinea pig cortex. However, contrary to this Goldstein *et al* [24] demonstrated central nervous system neuronal cell death after 60 min at temperatures as low as 40.5 °C.

The caveat to these studies is that they are acute, with sacrifice and histology a few days after the experiment. There are very few chronic long-term studies of the effect of raised temperatures. The key studies are perhaps by Seese *et al* and Okazaki *et al* [25, 26]. which demonstrate that d*T*  =  +2 °C is well tolerated in muscle. However, the long-term response to heat shock proteins and glial response in the CNS has not published. This is a challenge that needs to be explored in more detail by the biology community.

It is therefore perhaps unsurprising that the regulatory guidance is also limited Directive 93/42/EEC simply states ‘*Devices must be designed and manufactured in such a way as to remove or minimise as far as is possible*: 〈temperature rise〉’. The American Association of Medical Instrumentation (AAMI) recommend a limit of d*T*  =  +2 °C, which seems in keeping with the review above, i.e.  ⩽39 °C. So we work with the conservative limit of 2 °C above ambient as defined by the AAMI.

Given the uncertainties, it would be useful to have a tool to scientifically explore long-term chronic thermal increases and or thermal cycling. In particular, how this may result in inflammatory responses, probe rejection of even genetic mutation and tumour formation. Furthermore, if such a tool could be used *in situ* for neuroprosthetics, it could be used to optimise optical emission whilst maintaining surface temperatures below the Δ*T*  <  2 °C regulatory limit.

On-chip temperature sensors have been used to monitor human health for diseases diagnosis and treatment to monitor device temperatures and ensure they are maintained within desirable limits [27]. The adaptive multi-sensor CMOS system proposed by Huang *et al* [28] comprises different on-chip sensors including a temperature sensor which is using a pn junction to sense the body temperature. The low-cost CMOS thermal sensor chip for biomedical application presented by Lee *et al* is also employing a pn junction as temperature sensing element [29]. Crepaldi *et al* proposed a low power CMOS transistors based thermal sensing element for biomedical application [30]. The downside of using additional sensors is that they take additional surface space and need additional address architectures which may present difficulties to integration. Furthermore, it increases the complexity and thus cost of fabrication [31, 32]. Separate sensors for temperature sensing may cause a danger that failure in the sensor may provide inaccurate readings. Perhaps stating that the temperature rise is limited when the opposite is the case. In contrast, by utilizing the employed microphotonic emitter in an implantable device as its own sensor, the continued functionality of the device is intrinsically linked to its self-diagnosis.

There are a number of different forms of light emissive structures. However, as the light required for optogenetics is primarily blue, and must be high radiance, Gallium Nitride light emitting diodes (LEDs) are the primary technology. High radiance micro-photonics for neural stimulation have been successfully demonstrated in planar high radiance arrays by Soltan *et al* [33] and Berlinguer-Palmini *et al* [34]. More recently implementations in implantable probes have been demonstrated by Cao *et al* [15], Wu *et al* [16] and McAlinden *et al* [17].

As the surface temperature of a probe heats up, the junction temperature will also increase. The charge carrier generation in diodes is temperature dependent. As such, if the junction temperature (which is a function of surface temperature) increases, there will be a corresponding change in the LED carrier generation. In forward bias, the LED current exponentially increases with voltage, and thus small changes in temperature are not perceptible. However, in reverse bias, currents are dominated by leakage processes across the diode. In this case, the effect of voltage is still an order of magnitude greater than the effect of temperature change, but if current can be measured at a very stable voltage, then junction temperature can be ascertained.

**Figure 1.**
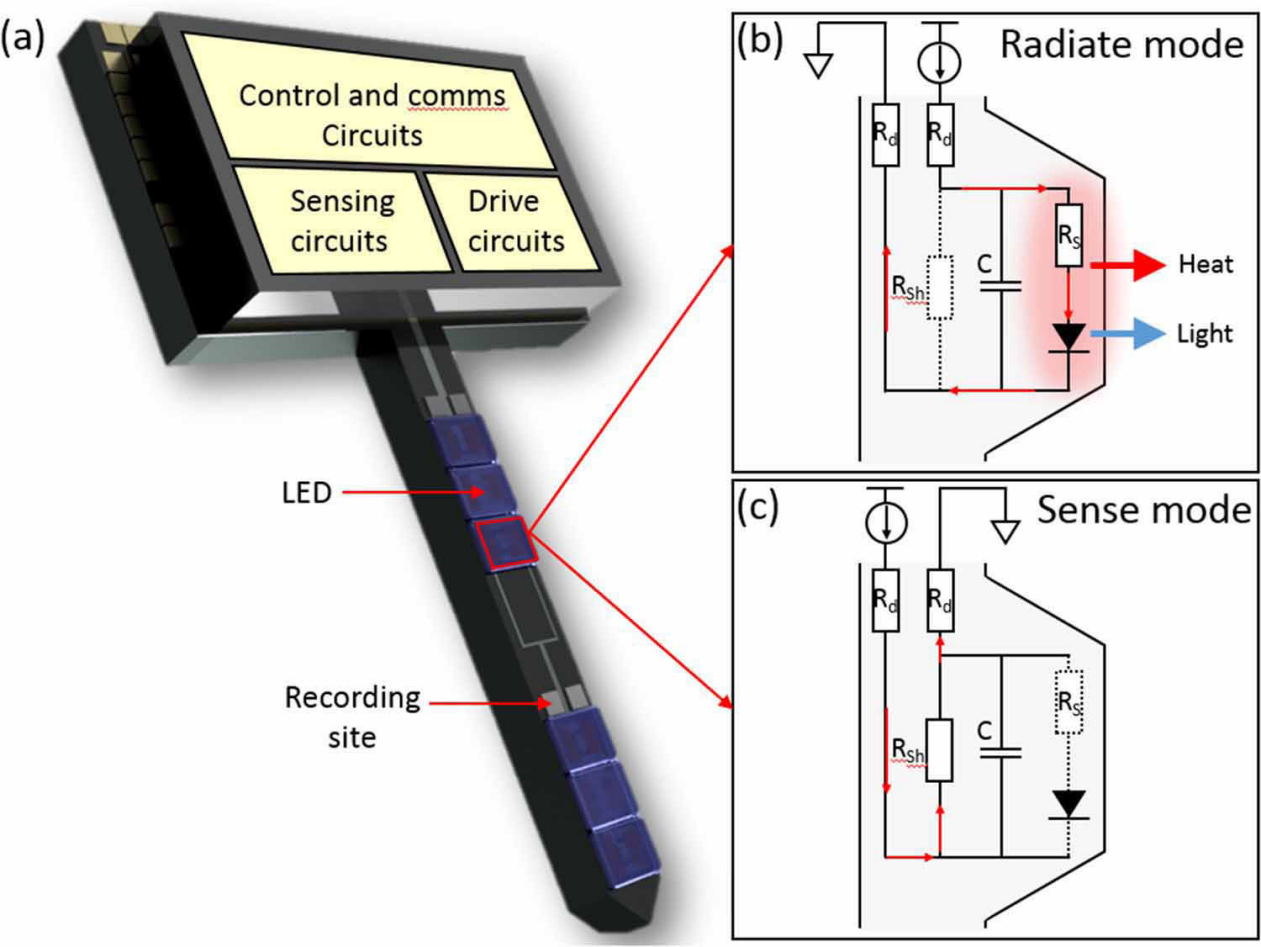
An illustration of optrode with LED and recording site on the shaft and driving and sensing circuits on the head (a) top view, (b) cross-section view showing forward biased LED and heat generation, (c) cross-section view showing reverse biased LED in thermal sensing mode.

In this work, LED as microphotonic emitter is presented as its own sensor on medical devices where a readout circuitry is developed to bias and measure the sensory parameter. Figure 1 shows a single penetrating active opto-electrode (optrode) and its general construction with inbuilt stimulation, recording circuits with a control logic unit [35]. Stimulation sites with micro/mini-LEDs for optical emitting and electrical recording sites with microelectrodes are placed along the shaft. The main issue here is the localized heating effects at the device surface caused by the shining LEDs which will be monitored using the designed temperature sensor. The proposed sensor utilizes the GaN LED itself as a sensor, combined with electronics designed using 0.35 *µ*m standard CMOS technology. The sensor circuits sit close to the LED to be operating in antiphase with optical stimulation. The proposed sensor is based on LED reverse current as temperature sensitive parameter (TSP). We have investigated several LEDs theoretically and experimentally in which their reverse current is linearly related to LED surface temperature (*T*_S_) [36]. Therefore, thermal variation around the reversely biased LED will change the reverse current. This current variation will be sensed, converted and amplified to a voltage signal which can be translated to temperature variations.

The paper structure is as follows: section 2 describes LEDs as a temperature sensor. The microelectronic structure of the sensor is explained in section 3. Section 4 provides the experimental methodology. The experimental results and discussion are given in section 5, and section 6 concludes the paper.

## 2. LEDs as temperature sensor

The operation of light emitting diodes is temperature dependent in both forward and reverse bias, as can be seen in figures 6(a) and (b). Jung *et al* [37] presented leakage current analysis of Gallium Nitride light emitting diodes in 2015. They showed that there were effectively 4 phases in the general conduction mechanism: Shunt resistance *R*_Sh_, parasitic diode, main diode, and sheet resistance *R*_S_. These have been presented in the equivalent circuit diagrams in figures 1(b) and (c), albeit with a single forward diode. Both the diode and shunt resistance operations are temperature dependent. However, as the diode current also varies exponentially with applied bias, any drift in bias could rapidly provide misleading results. In forward bias, there is a rapid transition between shunt resistance and diode limited current. As such, if the shunt resistance is to be utilised as a sensor, it would be preferable to do so in reverse bias.

The shunt resistance, which is responsible for the leakage has been presented by Jung [37] as having two primary mechanisms: variable range hopping (<300 K), and thermally-assisted multi-step tunnelling (>300 K). This description was also supported independently by Shan *et al* [38]. For implantable systems, the base temperature would be expected to be 310 K (i.e. 37 °C), and will increase during operation. As such, thermally-assisted multi-step tunnelling will be expected to be dominant. Such transport is characterised by the tunnelling of electronics from the valance band of the p-GaN to the conduction band of the n-GaN. The relationship between temperature and current is typical for diodes, i.e.

\begin{align} \newcommand{\e}{{\rm e}} \displaystyle {{I}_{{\rm leak}}}\propto {{{\rm e}}^{1/T}}.\nonumber \end{align}

However, there is a further caveat. The studies by Jung and Shan [37, 38] were for larger LEDs with surface passivation to prevent surface leakage. Such mechanisms are likely to be more considerable for smaller LEDs. Figures 6(b) and (d) presented in the results section show nonlinear increases in reverse current with applied bias. As such, it is important that any circuit measuring the temperature dependent leakage current ensures a stable applied bias. Furthermore, the temperature dependence of the LED current is effectively dependent on the temperature quantum well junction. In contrast, from the perspective of the sensor, it is the temperature of the surface of the probe which is of interest to ensure no negative biological impact.

**Figure 2.**
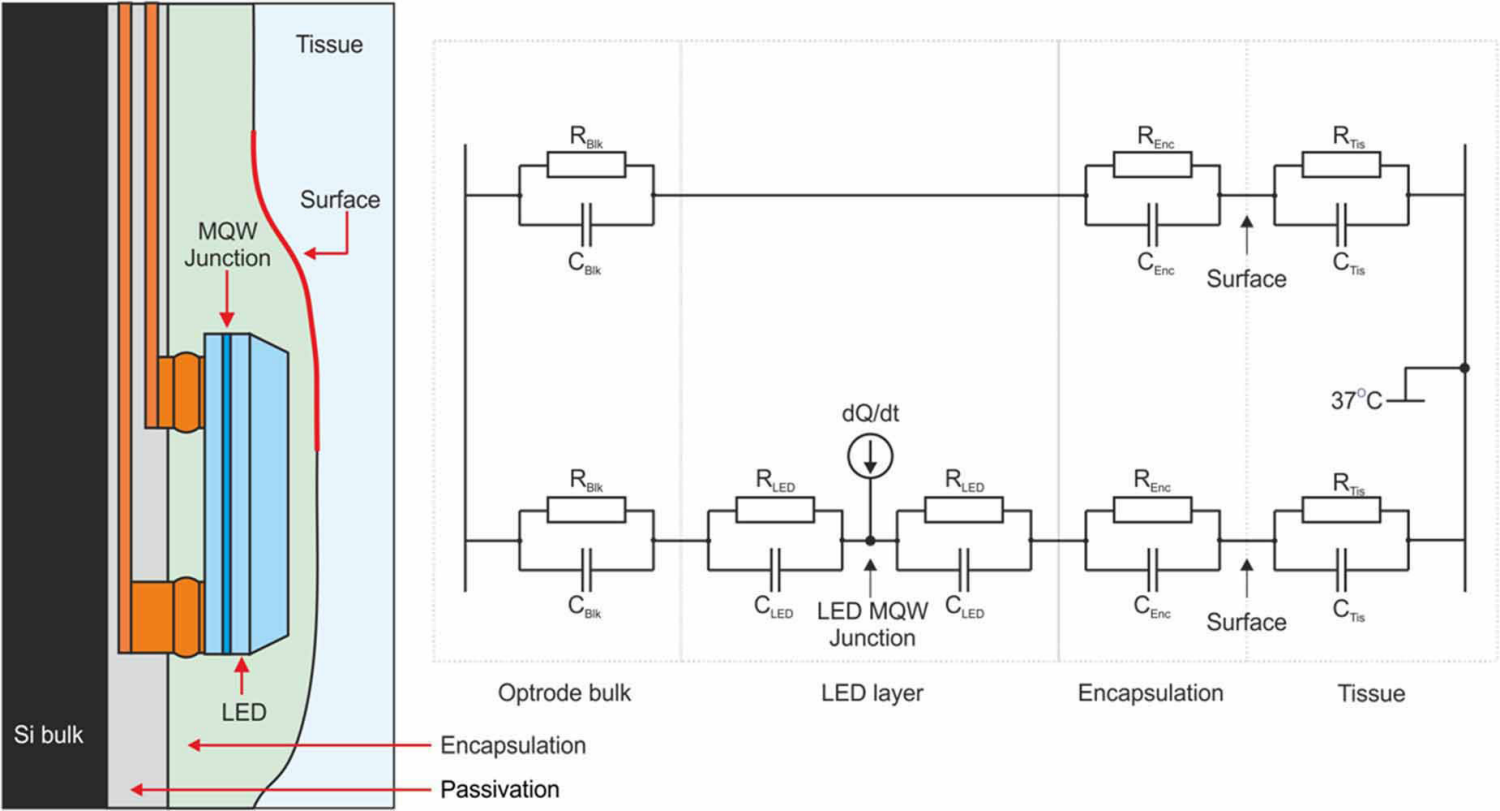
The thermal model of the optrode (Left) Physical structure around the LED part of the optrode. Dimensions and materials may change, e.g. silicon bulk, but the basic structure is common to all optrodes or implants incorporating emissive elements (right) a basic thermal model utilizing thermal resistances *R*, and heat capacity *C* for each of the dominant structures. Heat in occurs at the LED, and the boundary heat sink of the tissue is 37 °C.

Figure 2 (left) shows a simple thermal model of a Gallium Nitride LED in a typical embodiment on a thermal probe—e.g. as implemented in figures 4(c) and (d). Typically implantable optrode probes [15–17] have a silicon substrate with drive lines in a passivation layer (typically silicon dioxide) with bonded on LEDs and an encapsulation layer on top.

Figure 2 (right) shows a simple thermal model of the system based on passive diffusion. Thermal generation occurs due to resistances in both probe and LED and inefficiencies in light generation. We expect this to be primarily in the Multiple Quantum Well (MQW) junction of the diode. We can expect this to traverse the material and eventual the tissue through passive diffusion with each layer exhibiting both thermal resistance (*R*_Bulk_, *R*_LED_, *R*_Enc_, and *R*_Tis_) and heat capacity (*C*_Bulk_, *C*_LED_, *C*_Enc_, and *C*_Tis_). There will be additional effects of light absorption and vascular flows in the tissue.

We believe it is beyond the scope of this paper to accurately simulate this model. Rather we use it to understand the relationship between the temperature at the surface and that of the MQW junction. We then calibrate this empirically.

## 3. Microelectronic architecture

**Figure 3.**
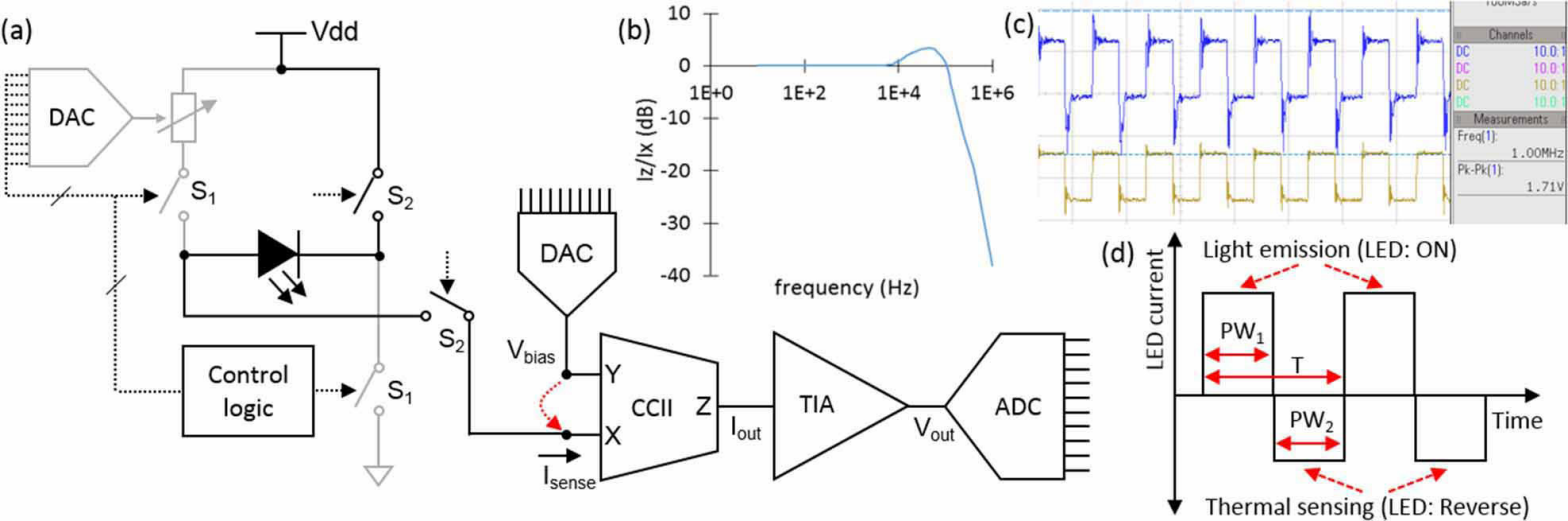
(a) Block diagram of the proposed temperature sensor where S1 switches close for light emission and only S2 switches close for temperature sensing. The bias voltage provided by DAC is transferred to X terminal in CCII which receives the LED current. A TIA converts and amplifies the current to large voltage signal, (b) the measured frequency response of CCII which shows the bandwidth is 130 kHz, (c) the time response of the pulsing LED with a clock signal of 1 MHz, (d) a timing diagram of the LED bias current including two phases.

A key issue with utilizing the LED as a temperature sensor is that both the voltage and temperature strongly affects the current. As such, to make a useful sensor, it is therefore important that a compact microcircuit can be developed which can sense the reverse current at a very stable voltage—i.e. stable with temperature, drift, and power supply fluctuations. An optimal circuit for achieving this is a second-generation current conveyor (CCII). It can be used to provide a precise bias voltage at the input (X), while receiving current using the same input terminal [39, 40]. The output of the current conveyor can then be transmitted to a transconductance amplifier. The subsequent output voltage can then be transferred to an analog to digital converter for digital transmission and analysis. Figure 3(a) depicts a block diagram of the developed microelectronic circuits. The CCII receives the bias voltage at the Y terminal from a digital to analog converter (DAC). It then copies the voltage at X terminal to bias the LED. In this design, *V*_X_ follows *V*_Y_ and the output current follows the input current (i.e. LED’s reverse current) received at X where the current transfer bandwidth is 130 kHz as illustrated in figure 3(b). A transimpedance amplifier (TIA) was designed to convert and amplify the output current of CCII with a gain of 5  ×  10^5^ V A^−1^. The circuit is robust against the power supply fluctuations as the measured PSRR is about 57 dB. Furthermore, the LED was pulsed with a frequency of 1 MHz to test the LED response. The LED switching response depicted in figure 3(c) shows the capability of the LED in fast On/Off switching at 1 MHz without affecting the time constant in the overall system.

The sensor electronics operate in antiphase with LED light emission, i.e. the first phase is light emission using forward biased LED through S1 switches while the intensity is controlled using pulse width modulation (PWM). The second phase, is temperatures sensing with reverse biased LED through S2 switches. The functionality of the sensor is explored by switching the LED from light emission to reverse mode using a continuous pulse. The generated heat after pulsing LED inside an isolated dark box was collected using IR camera as surface temperature. Figure 3(d) shows a timing diagram for light emission (PW_1_) and temperature sensing (PW_2_) phases.

The specifications of the designed temperature sensing system are listed in table 1. Temperature sensitivity of the sensor is related to LED type and its reverse current’s temperature dependency. For the given fixed bias voltage (−1.7 V) on the employed LEDs in the experiments, a temperature sensitivity at about 5 mV °C^−1^ can be achieved.

**Table 1.** Design specifications of the sensing system in *V*_bias_  =  −1.7 V for mini GaN LEDs.

Specification	Value
CMOS technology (*µ*m)	0.35 AMS
Supply voltage (V)	5
Power dissipation (Bias + CCII + TIA) (*µ*W)	260
Overall gain (V A^−1^)	5 × 10^5^
Temperature sensitivity (mV °C^−1^)	5–10
Sensitivity floor (°C)	0.2
Temperature range (°C)	25–65
PSRR (dB)	57 dB
Circuit Size (Bias + CCII + TIA) (*µ*m)^2^	133 × 64

## 4. Experimental methodology

In order to test our hypothesis, we have explored the temperature dependence of the reverse current on both micro-LEDs and mini LEDs. The former was custom developed according to [33, 41] with a 20 *µ*m diameter circular anode surrounding an 80 *µ*m square cathode. For comparison, we also used CREE DA2432 mini-LEDs which had dimensions of 240  ×  320 *µ*m. Exemplar images of these can be seen in figures 6(e) and (f).

**Figure 4.**
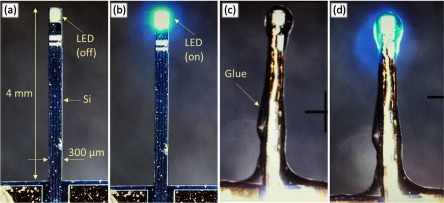
Micrograph of (a) off and (b) on non-encapsulated mini-LED, (c) off and (d) on encapsulated mini-LED mounted on a 4 mm silicon shank representing a typically penetrating probe for the brain cortex. Each of these probes was mounted on a PCB for handling (not shown).

We tested the micro-LED directly, but used the mini-LED to develop exemplar probes to determine their capability at measuring temperature in systems similar to the final embodiment. Probes were developed on silicon shafts which were 4 mm long, 300 *µ*m wide and 200 um thick. Titanium/Gold/Titanium metal tracks were deposited at a thickness of 20/200/20 nm and patterned. The metal tracks had a width of 30 um. The top Titanium on the bond pads was later removed and the Gold surface exposed. CREE DA2432 mini-LEDs were bonded on using silver epoxy (RS Pro Silver Vial Epoxy Conductive Adhesive) with a pick and place machine (FINEPLACER^®^ lambda from Finetech). The LEDs had a size of 240  ×  320 (*µ*m)^2^ and thickness of 140 *µ*m. Transparent silicone (NuSil MED-1000) was used to dip coat and encapsulate the LEDs. The encapsulated silicone had an average thickness of 50 *µ*m. Images of the developed probes can be seen in figure 4.

### 4.1. Calibration

**Figure 5.**
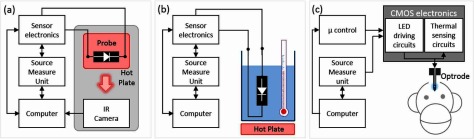
Diagram showing (a) the LED thermal characterization and sensor interface experimental setup inside an isolated dark box equipped with a hotplate. The sensor is connected to a source measure unit (SMU) and controlled using a computer. The temperature change is captured using an IR Optris PI camera, (b) the calibration setup using an encapsulated LED in water which is heated up using hotplate (c) the non-human-primate experimental setup in which an optrode with a passivated mini-LED is inserted into brain tissue. The microcontroller provides all needed control signals for LED driving and thermal sensing. SMU provides the bias voltages/currents and stores the output of the circuit.

Different experimental setups were developed to characterize the LED in air and water (saline) while connected to the sensor circuit. The measured data have been used to calibrate the probe for *in vivo* tests. Figure 5(a) shows the LED characterization setup inside an isolated dark box to explore the LED’s reverse current variation versus temperature in air when the LED is reversely biased. The isolated dark box guaranteed the measured current was due to the temperature change. The box was also temperature isolated to ensure the accuracy of the measured temperature. A hot plate of type VWR355 from VWR was placed under the LED to change the LED temperature in the range of 27 °C–60 °C while the LED temperature was measured using PI16O48T900 IR camera from Optris. The camera was set to a frame rate of 120 f s^−1^ to ensure the enough accuracy of the readings. The camera and desktop PC interface was through USB port and controlled through the Optris software. The sensor circuit was connected to a source measure unit (SMU) 2615B from Keithley and both the sensor and SMU were controlled using a computer program. Furthermore, the LED was pulsed between forward mode (LED on) and reverse mode (sensing) at room temperature, and the reverse current variation and surface temperature change have been measured during the LED reverse mode. For this experiment, the same setup in figure 5(a) excluding the hot late was used.

Figure 5(b) shows a diagram of the water experiment used to achieve the LED current and temperature variation inside water. The hotplate has been used to change the water temperature while a thermometer of type LO-tox from Brannan inside the water measured the temperature close to the LED. The water experiment started with the water at room temperature. The measured data from the air and water experiments have been used to calibrate the probe to use for thermal sensing in brain tissue. Figure 5(c) illustrates a diagram of the setup for non-human primate brain test which is described in the next section.

A conceptual thermal model for the probe is described in figure 2 and the experiments to perform a calibration are described in figures 5(a) and (b). The sensor fundamentally measures temperature dependent junction current (${{I}_{{\rm J}}}$), whereas we what is required is surface temperature $({{T}_{{\rm S}}})$. As such, we have devised two different calibration experiments: hotplate (figure 5(a)) and warm saline 37 °C  ±  1 °C (figure 5(b). The former can be used to assess surface temperature changes from a defined background temperature using infrared thermal imaging. However, this is only possible in air. In contrast saline experiments can be used to immerse the probe in a saline medium which is similar to that of nervous tissue, but we can only measure the bulk temperature.

Mathematically, we can express the relationship between junction current and junction temperature for both cases: hot plate ($\Delta {{I}_{{\rm JAir}}},\Delta {{T}_{{\rm JAir}}})$ and hot saline ($\Delta {{I}_{{\rm JWater}}},\Delta {{T}_{{\rm JWater}}}$) in terms of the change from the baseline as follows:

1 \begin{align} \newcommand{\e}{{\rm e}} \displaystyle \Delta {{I}_{{\rm JAir}}}={{\alpha }_{{\rm JAir}}}\cdot \Delta {{T}_{{\rm JAir}}}\nonumber \end{align}

2 \begin{align} \newcommand{\e}{{\rm e}} \displaystyle \Delta {{I}_{{\rm JWater}}}={{\alpha }_{{\rm JWater}}}\cdot \Delta {{T}_{{\rm JWater}}}.\nonumber \end{align}

Where $\Delta {{I}_{{\rm J}}}$ is the change in junction current with the change in junction temperature, $\Delta {{T}_{{\rm J}}}$. ${{\alpha }_{{\rm J}}}$ is the gradient of the relationship between $\Delta {{I}_{{\rm J}}}$ and $\Delta {{T}_{{\rm J}}}$ in air or water (or saline). However, we actually need the relationship between the sensed junction current $\Delta {{I}_{{\rm J}}}$ and changes in surface temperature $\Delta {{T}_{{\rm S}}}$ when the LED is pulsed. The hot plate experiment can provide this by pulsing the LED to have LED ON in forward bias (illumination) and LED OFF in reverse bias with a fixed overall background temperature. The changes in surface temperature can be measured through IR imaging simultaneous to changes in the junction current. This gives the following relationship:

3 \begin{align} \newcommand{\e}{{\rm e}} \displaystyle \Delta {{I}_{{\rm JAir}}}={{\alpha }_{{\rm SAir}}}\cdot \Delta {{T}_{{\rm SAir}}}.\nonumber \end{align}

Where $\Delta {{T}_{{\rm SAir}}}$ is the change in the surface temperature in air and ${{\alpha }_{{\rm SAir}}}$ is the gradient of the relationship between $\Delta {{I}_{{\rm J}}}$ and $\Delta {{T}_{{\rm S}}}$ in air. In this case, the temperature is not uniform throughout the probe. i.e. for (1) and (2) above, the probe is passively heated to a fixed temperature such that the bulk and surface are the same. In this case, both are heated to a baseline temperature and then the LED is turned on. The heat is thus generated in the junction which then transmits to the surface according to the model in figure 2, thus creating a gradient. This can be mathematically expressed as follows:

4 \begin{align} \newcommand{\e}{{\rm e}} \displaystyle {{\alpha }_{{\rm SAir}}}=\frac{\Delta {{I}_{{\rm JAir}}}}{\Delta {{T}_{{\rm SAir}}}}=\frac{\Delta {{I}_{{\rm JAir}}}}{\Delta {{T}_{{\rm SAir}}}}\cdot \frac{\Delta {{T}_{{\rm JAir}}}}{\Delta {{T}_{{\rm JAir}}}}.\nonumber \end{align}

But from (1):

5 \begin{align} \newcommand{\e}{{\rm e}} \displaystyle {{\alpha }_{{\rm JAir}}}=\frac{\Delta {{I}_{{\rm JAir}}}}{\Delta {{T}_{{\rm JAir}}}}.\nonumber \end{align}

So we can state:

6 \begin{align} \newcommand{\e}{{\rm e}} \displaystyle {{\alpha }_{{\rm SAir}}}={{\alpha }_{{\rm JAir}}}\cdot \frac{\Delta {{T}_{{\rm JAir}}}}{\Delta {{T}_{{\rm SAir}}}}.\nonumber \end{align}

As such ${{\alpha }_{{\rm SAir}}}$ respectively comprises of the fundamental LED relationships ${{\alpha }_{{\rm JAir}}}$ combined with the relationship between the change in junction temperature in air $\Delta {{T}_{{\rm JAir}}}$ with respect to surface temperature in water, $\Delta {{T}_{{\rm SAir}}}$. Our objective is to determine the surface temperature in water $\Delta {{T}_{{\rm SWater}}}$ from the junction current in water $\Delta {{I}_{{\rm JWater}}}$:

7 \begin{align} \newcommand{\e}{{\rm e}} \displaystyle \Delta {{I}_{{\rm JWater}}}={{\alpha }_{{\rm SWater}}}\cdot \Delta {{T}_{{\rm SWater}}}.\nonumber \end{align}

If we use the same logic as per (4)–(6) then we can state:

8 \begin{align} \newcommand{\e}{{\rm e}} \displaystyle \Delta {{I}_{{\rm JWater}}}={{\alpha }_{{\rm JWater}}}\cdot \left[ \frac{\Delta {{T}_{{\rm JWater}}}}{\Delta {{T}_{{\rm SWater}}}} \right]\cdot \Delta {{T}_{{\rm SWater}}}.\nonumber \end{align}

We can achieve the relationship ${{\alpha }_{{\rm JWater}}}$ using the experimental setup in figure 5(a). However, it is difficult to determine ${{\left| \Delta {{T}_{{\rm J}}} \right|}_{{\rm Water}}}/{{\left| \Delta {{T}_{{\rm S}}} \right|}_{{\rm Water}}}$. Thermal imager can only explore the surface and cannot see through water. Similarly, thermometers are bulk devices thus they cannot determine localized hotspots in the submillimeter scale. As such, we can utilize this relationship for air. The relationship between Δ*T*_SAir_ versus Δ*T*_JAir_ can be extracted using the measured currents from two different experiments which included LED pulsing and also hotplate to heat up the reverse biased LED.

### 4.2. Testing in non-human primate brain

Experiments were performed in non-human primate (NHP) brain to test the functionality for potential future clinical applications. A passivated optrode probe was inserted acutely into non-human primate (NHP) brain. Thermal recordings were obtained from non-human primates (macaca mulatta) as part of terminal experiments being conducted primarily for other research purposes. All procedures were approved by the local ethics committee at Newcastle University and performed under appropriate UK Home Office licenses in accordance with the Animals (Scientific Procedures) Act 1986. A ketamine/alfentanil infusion (0.1–0.6 mg kg^−1^ h^−1^ and 0.2 *µ*g kg^−1^ min^−1^ respectively) was used for anaesthesia, while core body temperature was maintained at 37–38° using a hot-air blanket (Bair Hugger, 3M). After a craniotomy and durotomy, LEDs were inserted to a depth of 2–3 mm into prefrontal regions of cortex using a stereotaxic manipulator. A diagram can be seen in figure 5(c).

## 5. Results and discussion

The proposed temperature sensing sub-system for optogenetics application utilises the incorporated LED as the primary sensor for detecting its own surface temperature. The implemented system clamps a reverse bias voltage with a high degree of accuracy across LED in order to differentiate between temperature variation and voltage variation where the voltage stability is within  ±5% of a target bias voltage. Temperature change can be accurately determined despite variations in power supply noise. A calibration process has been performed using the current–voltage-temperatures characteristics of the utilised LEDs in reverse bias to attain the surface temperature and current dependency of encapsulated and non-encapsulated LEDs. Also, different experiments are carried out to explore the LED’s junction and surface temperature relation. Furthermore, the sensor functionality is shown by conducting an experiment in non-human primate brain tissue.

**Figure 6.**
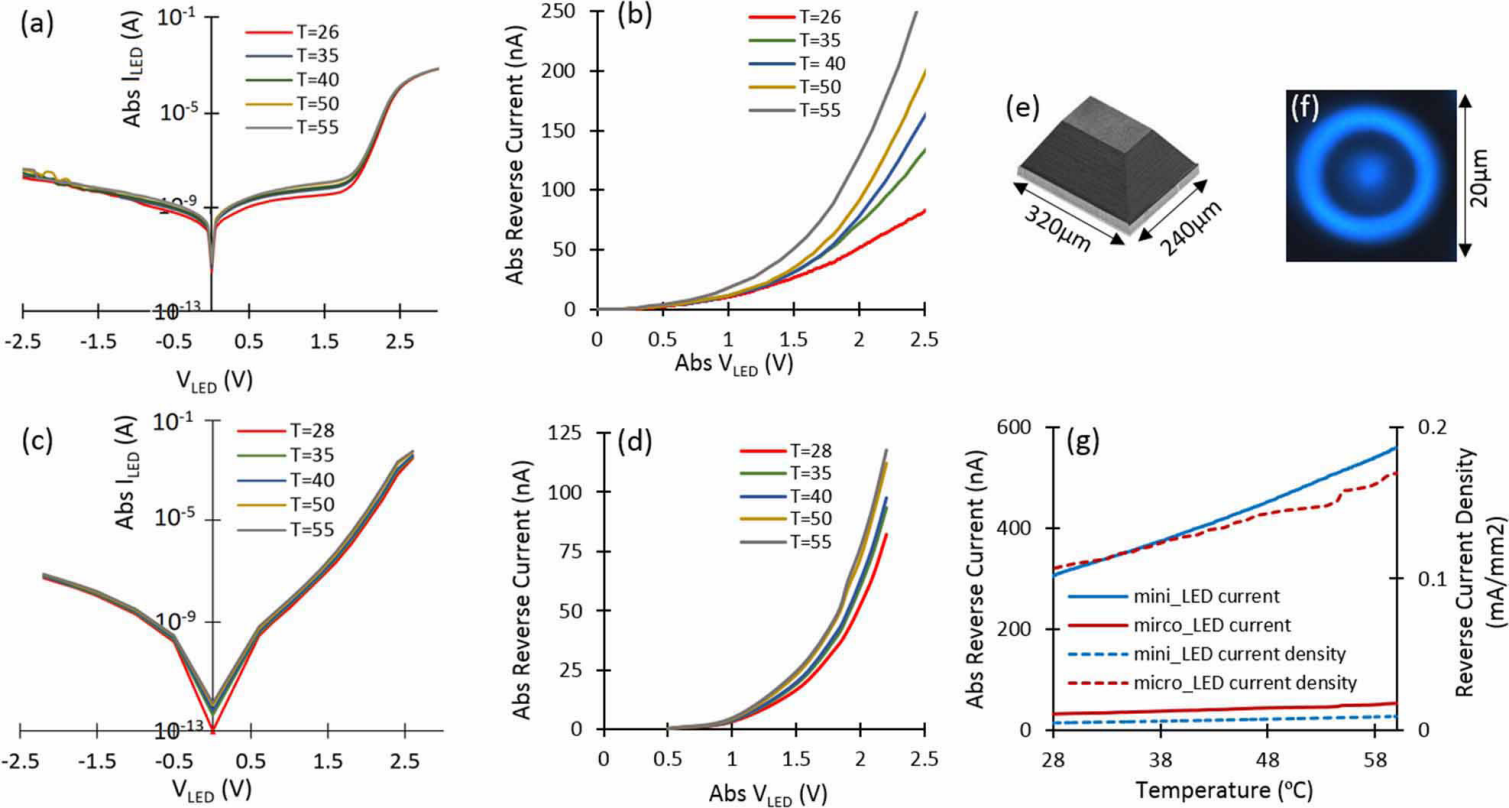
(a) The absolute current versus voltage in different temperatures for a mini-LED. (b) The absolute reverse current versus absolute voltage of a mini-LED in different temperatures, (c) the absolute current versus voltage in different temperatures for a micro-LED, (d) the absolute reverse current versus absolute voltage of a micro-LED in different temperatures, (e) and (f) an illustration of the employed mini-LED and micro-LED, (g) absolute reverse current and reverse current density versus temperature for mini-LED and micro-LED when they are biased using  −1.7 V.

We have experimentally explored standard Gallium Nitride (GaN) LEDs characteristics to achieve the relation between LED’s reverse current and temperature. Figure 6(a) shows the *I*–*V* characteristics of a mini-LED at different temperatures. Figure 6(b) shows the absolute reverse current of mini-LED versus absolute voltage in different temperatures to explore these three parameters’ relationship. The results show the current changes exponentially and considerably due to the bias voltage variations more than the temperature variations. Therefore, it can be very challenging to determine the LED’s temperature in reverse bias based on the reverse current. This means the LED needs to be interrogated at a fixed reverse bias which does not deviate in time or due to noise. Figure 6(c) shows the *I*–*V* characteristics of a micro-LED at different temperatures. Figure 6(d) shows the absolute reverse current of micro-LED versus absolute reverse voltage in different temperatures. The illustration of mini and micro LEDs are shown in figures 6(e) and (f).

Figure 6(g) shows the absolute value of reverse current and current density versus temperature for a mini-LED and micro-LED when they are reversely biased with  −1.7 V. The current of the biased LEDs was measured while the temperature was rising using hotplate. As can be seen in the results, there is a good linearity between reverse current and temperature which shows that the reverse current can be used as a semi-linear TSP. It’s clear that bigger LEDs will have more current variation for specific temperature change. As noted in the introduction, the reverse leakage profile is very different between commercial mini-LEDs and custom micro-LEDs. For this reason, we plot (a)–(d) in absolute current rather than current density. We believe the reasons for this lie with both differences in structural configuration and surface to bulk ratios.

### 5.1. Experimental results for encapsulated and non-encapsulated LEDs

**Figure 7.**
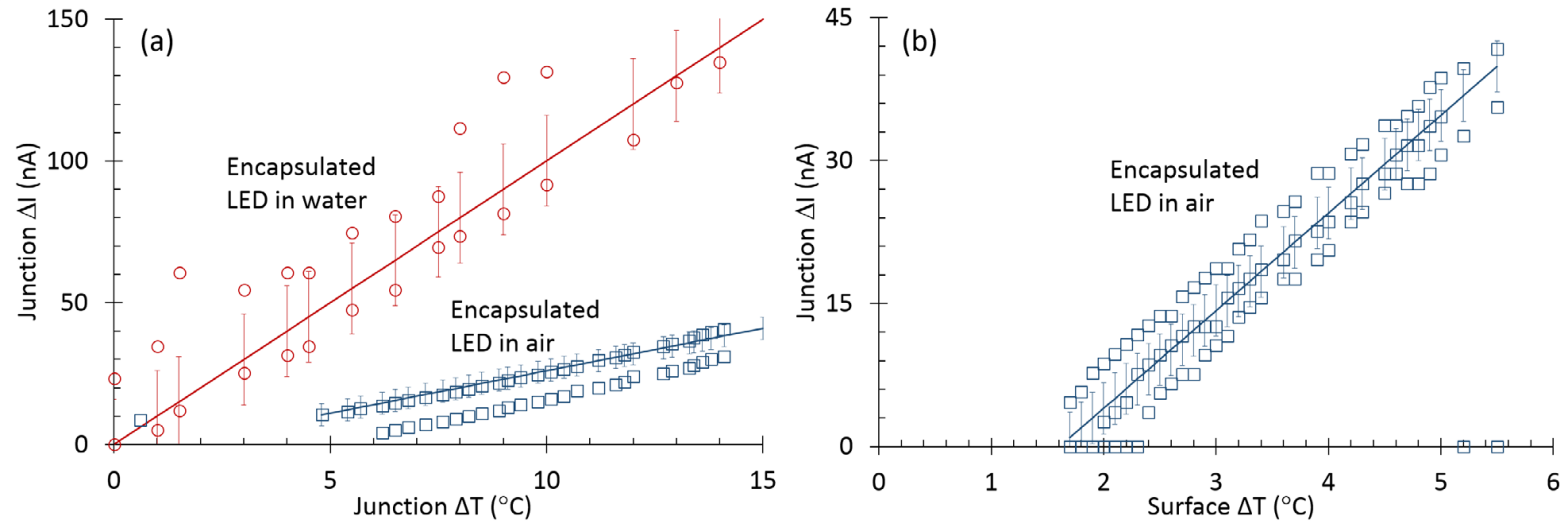
Measurement results achieved from the encapsulated LED as (a) junction current change versus junction temperature change in water and air by using a hotplate to change the temerature (b) junction current change versus surface temperature change in air when the LED was driven using a 2.5 mA pulse at a base temperature of 27 °C.

The Hotplate and warm saline experiments were performed based on the setups in figures 5(a) and (b) to assess the relationship between the junction current ${{I}_{{\rm J}}}$ and the junction temperature *T*_J_ in air and Saline. Figure 7(a) shows the junction current change versus junction temperature change for an encapsulated LED in air and water where the junction current change is linear within 15 °C junction temperature variation. On the other hand the LED pulsing experiment was carried out to achieve the LED surface temperature in air. Figure 7(b) shows the junction current change versus surface temperature change for an encapsulated LED in air which shows a linear relation for 6 °C temperature change. Values for the gradients are shown in table 2 below. There was some offsets from zero in our measurements due to drift in our experimental setup, but the gradients themselves are repeatable as shown in the multiple scatter plots.

**Table 2.** Extracted regression analysis parameters for the measured data in figures 7 and 8.

Figure	Characteristic	Value	*σ*
7(a)	$\frac{\Delta {{I}_{{\rm JAir}}}}{\Delta {{T}_{{\rm JAir}}}}$	10	18
7(a)	$\frac{\Delta {{I}_{{\rm JWater}}}}{\Delta {{T}_{{\rm JWater}}}}$	3.8	3.8
7(b)	$\frac{\Delta {{I}_{{\rm JAir}}}}{\Delta {{T}_{{\rm SAir}}}}$	10.3	2.7
8(a)	$\frac{\Delta {{T}_{{\rm SAir}}}}{\Delta {{T}_{{\rm JAir}}}}$	0.34	0.3
8(b)	$\frac{\Delta {{I}_{{\rm JWater}}}}{\Delta {{T}_{{\rm JWater}}}}$	29.4	3

**Figure 8.**
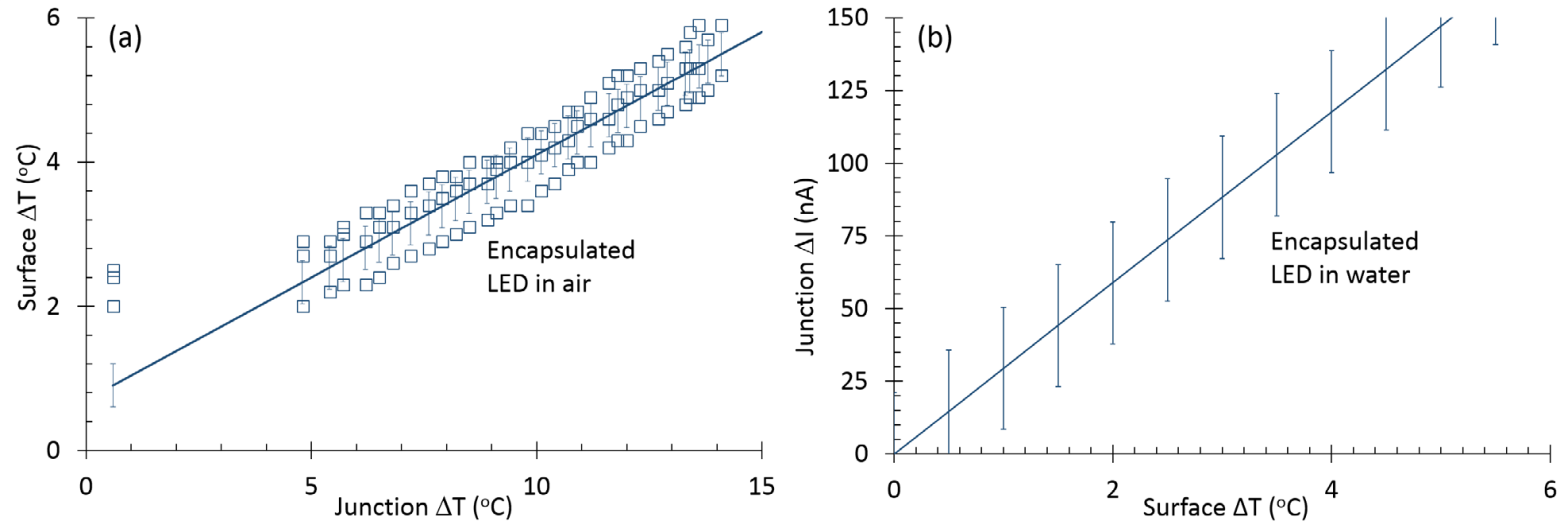
Extracted data for the encapsulated LED showing (a) the relationship between the LED surface and junction temperature change in air (b) the LED junction current change versus surface temperature change in water.

Figure 8(a) shows the relationship between the junction and surface temperature in air. From figures 7(a), (b) and 8(a), a relationship between the surface temperature and the junction current can be derived. This is shown in figure 8(b). The derivation for this has been described above in equations (1)–(8). The total error is derived from summing the standard errors from each of the gradient relationships. The extracted regression analysis parameters are summarized on table 2.

**Figure 9.**
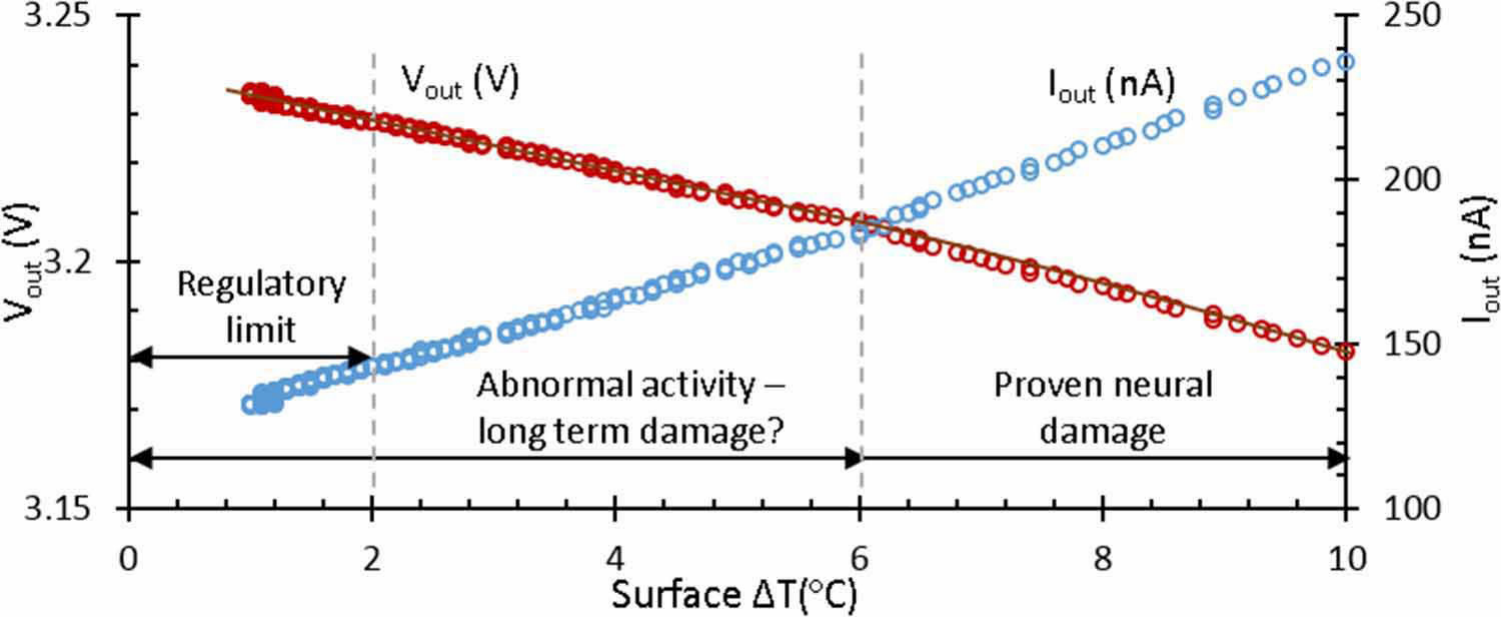
Measured sensor output current and voltage change versus surface temperature change when the encapsulated LED was driven using a 7.5 mA pulse.

Figure 9 shows the CCII output current and also the sensor output voltage versus Δ*T*_S_ which is likely linear. For this experiment, a long 7.5 mA pulse is applied to the LED for a few minutes. During pulse-On, the surface temperature increases about 25 °C (from 27 °C to 52 °C) and saturates. In the reverse sensing phase, the LED is reverse biased, and the temperature starts to decrease. Consequently, LED reverse current, the CCII output current and the sensor output voltage change. The change in LED current for 25 °C temperature variation was conveyed with unity gain to the CCII output while the LED was biased using the CCII. The output current was converted and amplified using TIA to about 200 mV output voltage which gives 8 mV °C^−1^ temperature sensitivity for the sensor.

### 5.2. Experimental results from non-human primate brain

**Figure 10.**
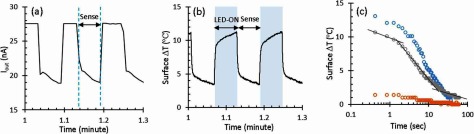
(a) and (b) Exemplar results for the output current of CCII and surface temperature variation during LED-illumination and sensing phases, (c) surface temperature versus time during sensing phase when three different pulses with different amplitudes (2 mA, 4 mA and 8 mA) are applied to the LED inserted in monkey’s brain.

The final experiment was to explore the efficacy of the probe in a real world scenario close to human neuroprosthetics. The probe was inserted into non-human primate brain according to figure 5(c). The LED was driven in two phases—LED illumination, and reverse sensing while the sensor was used to bias the LED and measured the reverse current (figure 10(a)). The calibration method was then applied to the measured current to extract the LED’s surface temperature as shown in figure 10(b). As can be seen in the sensing phase, the bigger current is related to higher temperature. This means the LED bias and the applied pulse will have different current and then temperature change. To see the effect of pulse amplitude, three different long pulses with different amplitudes (2 mA, 4 mA and 8 mA) were applied to the LED, and the output current was measured during reverse sensing phase. The related surface temperature was extracted for each experiment as shown in figure 10(c). The results show that the surface temperature changes after the LED illumination while this generated surface temperature is decreasing during the *reverse-sensing* phase. The results also show that the more intense LED radiation results in larger thermal emission which takes a longer time to cool down.

The thermal decay profile is expected to be exponential according to the passive RC network model described in figure 2. As such straight lines have been provided in the centre data of figure 10(c) to show 3 dominant time constants, which we assume to relate to the optrode bulk, LED, and encapsulation.

## 6. Conclusion

An LED-based temperature sensor is designed in standard 0.35 *µ*m CMOS technology to detect the change of temperature at the surface of the implanted LEDs in biomedical applications like optogenetics. This is to prolong the lifespan of the implants and prevent tissue damage as the increased heat due to LED shining can damage LED bonding and neural tissue. To achieve this the designed sensor measures the LED reverse current as the experimental results for different GaN LEDs show that the LED reverse current can be employed as a reliable temperature sensitive parameter to detect the LED junction temperature change. Furthermore, the employed calibration method enables extracting the LED surface temperature change in air and water which occurs after LED illumination, and it’s our interest. Therefore, by measuring the surface temperature change we can control the heating issues after LED illumination. The designed CMOS temperature sensor consists of a second generation current conveyor to bias the LED, receive and convey the reverse current to a high gain transconductance amplifier which converts and amplifies the signal. The sensor output signal can be then digitised and translated into a temperature change. This proposed method of temperature sensing is area efficient by eliminating area consuming blocks which are usually used for temperature sensing in implantable systems. This means the danger of failure because of the other devices can be decreased here.
